# Transcranial Direct Current Stimulation (tDCS) for the Treatment of Hemolacria Comorbid With Psychiatric and Somatic Symptoms: A Case Report

**DOI:** 10.1002/ccr3.72787

**Published:** 2026-05-24

**Authors:** Roghayeh Mohammadi, Ahmad Alipour

**Affiliations:** ^1^ Department of Psychology Payame Noor University Tehran Iran

**Keywords:** anxiety, depression, Hemolacria, insomnia, tDCS

## Abstract

Hemolacria is a rare condition characterized by bloody tears. Its etiology is often multifactorial, but functional/psychosomatic mechanisms have been proposed when organic causes are excluded. A 48‐year‐old woman with a history of multiple traumatic brain injuries (coma in 2008, car accident in 2017) presented with refractory generalized anxiety disorder, major depressive disorder, insomnia disorder, and treatment‐resistant hypertension. Since 2022, she developed bilateral hemolacria with a baseline frequency of 4–5 episodes daily despite extensive negative ophthalmological, neurological, hematological, and imaging workups. She had been on stable doses of sertraline 100 mg/day, amlodipine 10 mg/day, losartan 100 mg/day, and intermittent zolpidem for at least 6 months. Anodal transcranial direct current stimulation (tDCS) targeting the left dorsolateral prefrontal cortex (L‐DLPFC; anode F3, cathode Fp2) was administered for 27 sessions over 61 days (1–2 mA, 20 min/session, 5 days/week). No concurrent medication changes were made during or after the intervention. Marked clinical improvement began around session 12. By session 27, standardized scores showed remission of symptoms (HAM‐D‐17 from 26 to 4, HAM‐A from 34 to 6, PSQI from 16 to 5), office blood pressure normalized to 125–135/80–85 mmHg, and hemolacria frequency decreased to one minor episode every 43 days. All improvements were sustained at the 3‐month follow‐up without further tDCS. This single‐case, open‐label report describes concurrent reductions in psychiatric, cardiovascular, and hemolacria symptoms that occurred in temporal association with anodal tDCS over the L‐DLPFC in a treatment‐refractory patient after organic causes had been excluded. Given the uncontrolled design, causality cannot be established; alternative explanations including placebo response, non‐specific effects of clinical attention, regression to the mean, or spontaneous remission cannot be excluded. Randomized controlled trials are warranted to investigate the potential efficacy and mechanisms of tDCS in functional hemolacria.

## Key Clinical Message

In this single case, marked reductions in refractory hemolacria and comorbid severe anxiety, depression, insomnia, and hypertension were observed in temporal association with anodal tDCS over the L‐DLPFC, after organic causes had been excluded. Sustained improvements across domains were maintained at the 3‐month follow‐up after 27 sessions; however, given the open‐label, uncontrolled design, causality cannot be established and placebo or non‐specific effects cannot be excluded.

## 1 Introduction

Hemolacria, the presence of blood in tears, is a rare and dramatic clinical phenomenon that has intrigued physicians since antiquity and continues to cause profound distress for affected patients and their families [1, 2]. Also referred to as bloody epiphora, dacryohemorrhea, or simply “crying blood,” the condition is reported more frequently in females and may present unilaterally or bilaterally [3]. Recognized etiologies are diverse and include local ocular or lacrimal pathology (e.g., conjunctival telangiectasia, tumors, dacryoliths, retrograde epistaxis), trauma, infections, coagulopathies, vicarious menstruation, organophosphate poisoning, severe epistaxis, and hypertensive crises [3, 4].

However, in a subset of extensively evaluated cases, comprehensive ophthalmological, otorhinolaryngological, hematological, and neuroimaging investigations fail to identify any structural or systemic abnormalities [5, 6, 7]. In these instances, a functional or psychosomatic mechanism has been proposed, particularly when hemolacria arises or worsens in the context of severe psychological stress, anxiety, or depressive disorders [8, 9]. Consistent with this hypothesis, prior reports have described complete or near‐complete resolution of bloody tears following pharmacologic treatment of comorbid psychiatric conditions with anxiolytics, antidepressants, or beta‐blockers that attenuate sympathetic hyperarousal [8, 9, 10].

Transcranial direct current stimulation (tDCS) is a safe, well‐tolerated, non‐invasive neuromodulation technique that modulates cortical excitability and functional connectivity via weak direct currents delivered through scalp electrodes [11, 12, 13, 14]. Anodal stimulation of the left dorsolateral prefrontal cortex (L‐DLPFC) has consistently shown antidepressant and anxiolytic effects in randomized controlled trials, along with beneficial effects on sleep architecture and autonomic cardiovascular regulation [12, 13, 14, 15, 16].

Emerging evidence also suggests that anodal tDCS may improve endothelial function and lower blood pressure in patients with resistant hypertension, potentially through modulation of central autonomic networks.

To our knowledge, no prior report has explored neuromodulation as a treatment for hemolacria. Here, we present the first documented case of refractory hemolacria co‐occurring with treatment‐resistant major depressive disorder, generalized anxiety disorder, insomnia disorder, and poorly controlled hypertension. Following 27 sessions of anodal tDCS over the L‐DLPFC, marked reductions in symptom severity across these domains were observed in temporal association with the intervention, after thorough exclusion of organic causes. This single‐case observation is hypothesis‐generating and suggests that targeted cortical neuromodulation may be associated with improvement in both psychiatric and somatic symptoms in selected treatment‐refractory patients, although causality cannot be inferred from the present uncontrolled design.

## 2 Case History/Examination

The patient was a 48‐year‐old married woman with middle‐school education, residing in Maragheh, a medium‐sized urban city in northwestern Iran. Her past medical history was significant for two episodes of severe traumatic brain injury.

The first injury occurred in 2008 as a result of an accidental fall from height (slipping on stairs at home), which led to an initial loss of consciousness lasting approximately 5–6 h, followed by a six‐month period of coma. She experienced post‐traumatic amnesia and required prolonged intensive care management. Available medical records from that time reportedly documented diffuse cerebral edema and small bilateral frontal contusions (detailed original imaging not available for review).

The second injury took place in 2017 in a high‐speed motor vehicle accident, resulting in transient right‐eye blindness lasting 14 days (attributed to post‐traumatic optic neuropathy) along with symptoms of concussion.

Psychiatric symptoms began insidiously following the 2008 injury and became markedly more severe after the 2017 accident. The predominant complaints included persistent low mood, anhedonia, excessive and uncontrollable worry, restlessness, irritability, impaired concentration, profound fatigue, and severe sleep disturbance. These symptoms fulfilled DSM‐5 criteria for major depressive disorder and generalized anxiety disorder [17].

**FIGURE 1:**
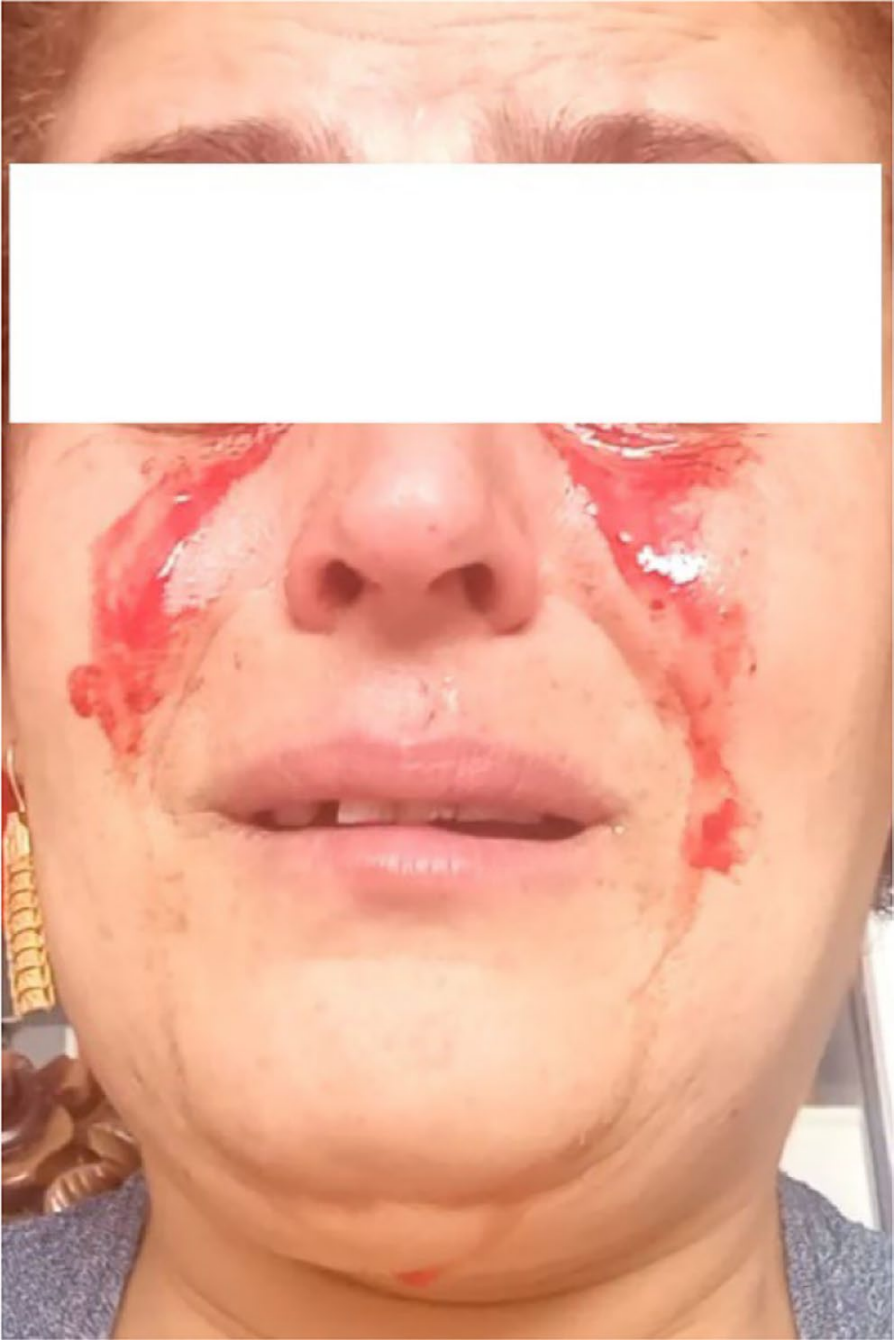
Bilateral hemolacria before initiation of tDCS treatment (April 2024). Frank blood is visible mixing with tears in both eyes. (Written informed consent was obtained from the patient for publication of this image, including permission to use masked or cropped versions in scientific publications.).

Subsequently, she developed treatment‐resistant hypertension with repeatedly documented systolic blood pressure readings in the range of 180–220 mmHg. In 2022, she began experiencing bilateral hemolacria (bloody tears). Figure 1 shows the typical appearance of bilateral hemolacria in this patient prior to initiation of tDCS treatment (April 2024), with frank blood visibly mixing with tears in both eyes. The photograph was taken by the patient herself at home.

At the time of initial assessment for tDCS (April 2024), she had been receiving stable pharmacotherapy for at least six months, consisting of sertraline 100 mg/day, amlodipine 10 mg/day, losartan 100 mg/day, and intermittent zolpidem as needed for sleep. No changes were made to this regimen during the tDCS course or during the follow‐up period.

The patient reported significant functional impairment in daily activities and marked subjective distress due to the combination of psychiatric symptoms, poorly controlled blood pressure, and frequent episodes of visible bloody tears (patient‐reported baseline frequency of 4–5 episodes per day). Hemolacria episodes were documented solely through the patient's daily symptom diary; no episodes were directly observed by the clinical team during any routine visits. To enhance clarity regarding the chronological sequence of events, key clinical milestones are summarized in Table 1.

**TABLE 1:** Timeline of key clinical events.

Year/Period	Event
2008	Severe traumatic brain injury (fall from height); loss of consciousness 5–6 h, followed by 6‐month coma; diffuse cerebral edema and small bilateral frontal contusions
2017	High‐speed motor vehicle accident; transient right‐eye blindness (14 days) and concussion
Post‐2008 (insidious onset); worsened post‐2017	Onset and progressive worsening of major depressive disorder, generalized anxiety disorder, and insomnia disorder
Post‐2017	Development of treatment‐resistant hypertension (systolic BP frequently 180–220 mmHg)
2022	Onset of bilateral hemolacria (baseline frequency: 4–5 episodes per day)
At least 6 months prior to April 2024	Stable pharmacotherapy (sertraline 100 mg/day, amlodipine 10 mg/day, losartan 100 mg/day, intermittent zolpidem)
April 2024	Baseline assessment for tDCS: HAM‐D‐17 = 26, HAM‐A = 34, PSQI = 16; frequent BP > 180/110 mmHg; Figure 1 (bilateral hemolacria)
12 April – 11 June 2024 (61 days)	27 sessions of anodal tDCS targeting L‐DLPFC (1–2 mA, 20 min/session, 5 days/week); no medication changes
Around session 12	Onset of noticeable clinical improvement
11 June 2024 (end of tDCS)	HAM‐D‐17 = 4, HAM‐A = 6, PSQI = 5; BP 125–135/80–85 mmHg; hemolacria reduced to ~1 minor episode every 43 days
September 2024 (3‐month follow‐up)	Sustained reductions in symptoms: HAM‐D‐17 = 3, HAM‐A = 5, PSQI = 4; BP 128–132/78–84 mmHg; no hemolacria episodes since end of treatment

## 3 Differential Diagnosis, Investigations and Treatment

The differential diagnosis of hemolacria encompasses a wide range of organic and systemic causes that must be systematically excluded before attributing the condition to a functional or psychosomatic mechanism. Recognized organic etiologies include local ocular and lacrimal pathologies (such as conjunctival telangiectasia, tumors, dacryoliths, severe conjunctivitis, or lacrimal gland abnormalities), retrograde epistaxis secondary to nasal or sinus disease, vascular malformations, coagulopathies, trauma to the lacrimal apparatus, vicarious menstruation (ocular vicarious menstruation), organophosphate poisoning, and episodes of severe hypertensive crisis with associated retinopathy or microvascular damage. In addition, factitious disorder (Munchausen syndrome) was considered in the differential diagnosis, given reports of hemolacria as a presentation of factitious bleeding. In this patient, the bilateral and recurrent nature of the bloody tears, combined with the absence of any acute traumatic or infectious trigger, necessitated a thorough multidisciplinary evaluation to rule out these possibilities.

**FIGURE 2:**
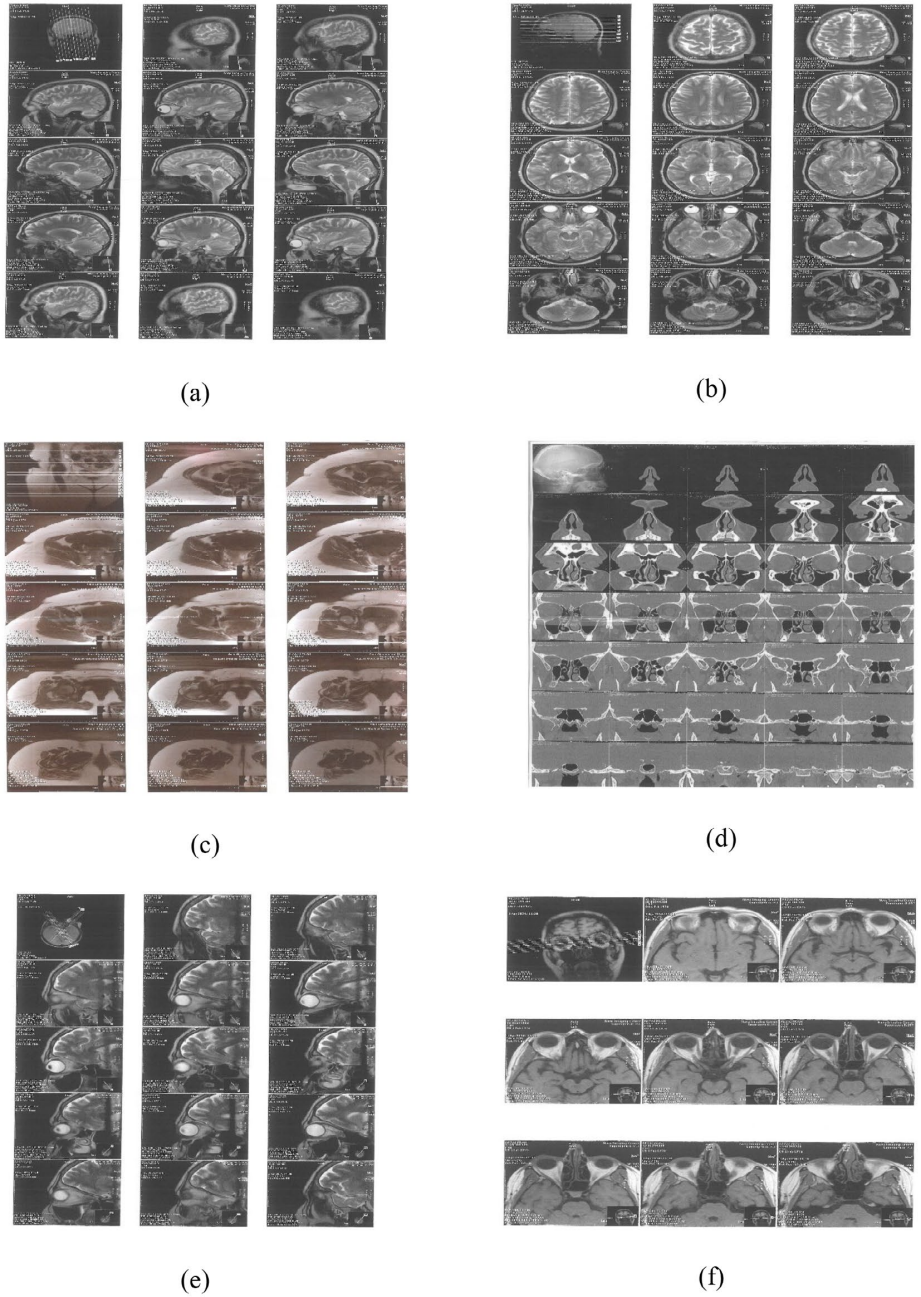
Normal diagnostic imaging findings prior to tDCS treatment (March–April 2024). (a, b) Contrast‐enhanced axial T1‐weighted brain MRI. (c, d) Orbital CT scans. (e, f) Nasal endoscopy views. No abnormalities were detected.

Between 2022 and April 2024, the patient underwent extensive investigations to exclude organic causes. These included repeated detailed ophthalmological examinations (slit‐lamp biomicroscopy, funduscopy, tear film break‐up time, Schirmer test, and lacrimal duct probing), nasal endoscopy to assess for retrograde epistaxis or sinus pathology, contrast‐enhanced MRI of the brain and orbits, CT angiography of the head and neck vasculature, complete coagulation profile (PT, aPTT, INR, bleeding time, platelet function assay), full blood count, comprehensive biochemistry panel, and thyroid function tests. All results were within normal limits or considered non‐contributory to the presentation of recurrent bilateral hemolacria (Figure 2). No structural, vascular, hematological, or lacrimal abnormality was identified. Vicarious menstruation was deemed unlikely due to the lack of clear cyclical association with menses, and factitious disorder was not supported after careful clinical evaluation.

Following the exclusion of organic pathology, formal psychiatric evaluation confirmed the diagnoses of treatment‐resistant major depressive disorder, generalized anxiety disorder, and insomnia disorder based on DSM‐5 criteria. At baseline assessment in April 2024, symptom severity was in the severe range: HAM‐D‐17 score of 26, HAM‐A score of 34, and PSQI score of 16. Psychiatric diagnoses and standardized rating scales were administered by a qualified clinical psychologist, while ophthalmological, neuroimaging, and otorhinolaryngological evaluations were performed by board‐certified specialists. Antihypertensive pharmacotherapy was managed by an internal medicine physician.

Given the patient's severe uncontrolled hypertension (systolic BP frequently 180–220 mmHg), pre‐session blood pressure screening was conducted before each tDCS session using a calibrated automated sphygmomanometer. Sessions were postponed if systolic blood pressure exceeded 200 mmHg, and the patient was monitored for any neurological or cardiovascular symptoms. No adverse events occurred.

The patient had been maintained on a stable medication regimen for at least six months, consisting of sertraline 100 mg/day, amlodipine 10 mg/day, losartan 100 mg/day, and intermittent zolpidem as needed for sleep, with no changes introduced during the subsequent intervention or follow‐up period.

Given the refractory psychiatric symptoms, poorly controlled hypertension, and functionally impairing hemolacria despite optimized pharmacotherapy, anodal tDCS targeting the left dorsolateral prefrontal cortex (L‐DLPFC) was offered as an adjunctive neuromodulation treatment. The protocol (detailed in Table 2) involved 27 sessions delivered over 61 calendar days using a CE‐marked NeuroConn DC‐Stimulator PLUS device (anode at F3, cathode at Fp2; initial intensity 1.0 mA for 7 sessions, then 2.0 mA for the remaining 20 sessions; 20 min per session, 5 days per week). The tDCS sessions were administered by a trained clinician. No adverse effects were reported throughout the course of treatment.

**TABLE 2:** Detailed tDCS protocol.

Parameter	Specification
Electrode positions (10–20 EEG system)	Anode: F3 (left dorsolateral prefrontal cortex); Cathode: Fp2 (right supraorbital)
Electrode size	35 cm^2^ (5 × 7 cm) saline‐soaked sponge electrodes
Current intensity	Sessions 1–7: 1.0 mA; Sessions 8–27: 2.0 mA
Ramp‐up/ramp‐down	30 s
Session duration	20 min
Frequency	5 days/week (Monday–Friday)
Total sessions	27 sessions
Treatment period	12 April 2024–11 June 2024 (61 calendar days)
Schedule clarification	Sessions were delivered on weekdays; occasional interruptions due to national holidays and patient availability resulted in 27 sessions completed over 61 calendar days (no sessions intentionally missed; protocol adhered to planned schedule with minor non‐consecutive adjustments)
Device	NeuroConn DC‐Stimulator PLUS (neuroCare Group, Ilmenau, Germany)
Concomitant medications	Unchanged throughout treatment and follow‐up
Adverse effects	None reported (no tingling, itching, burning sensation, headache, or skin irritation at electrode sites)

Hemolacria frequency was prospectively recorded by the patient in a daily symptom diary reviewed at each session. No episodes of hemolacria were directly observed by the clinical team during routine visits. The assessment relied primarily on patient self‐report through the diary, with illustrative clinical photographs obtained at selected time points to document the condition before and after treatment.

Standardized psychiatric rating scales were administered at baseline, periodically during treatment, at the end of the protocol, and at the 3‐month follow‐up. Rating‐scale assessments were performed independently of the tDCS operator.

## 4 Conclusion and Results

**FIGURE 3:**
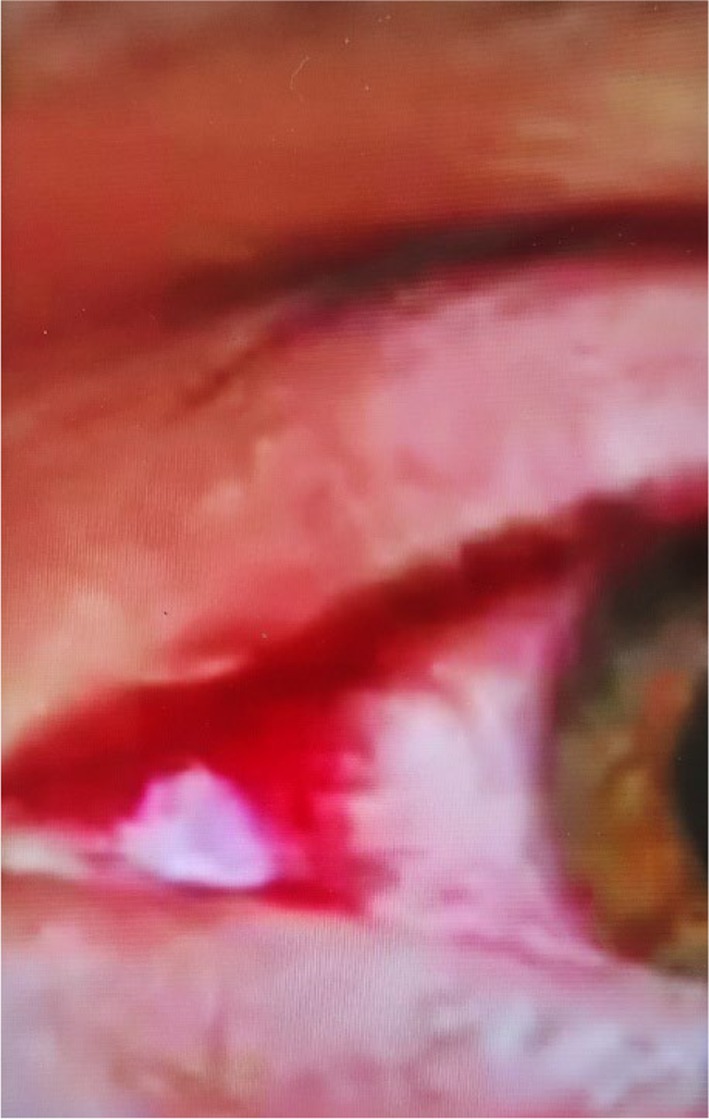
Markedly reduced unilateral hemolacria observed approximately 43 days after completion of the 27‐session tDCS protocol (July 2024). Only minimal blood staining is visible in the right eye.

Clinical improvement became clearly evident around the 12th session of tDCS, with the patient reporting a noticeable reduction in anxiety intensity, a marked decrease in the frequency and severity of bloody tear episodes, and subjective improvements in mood and sleep continuity. These patient‐reported changes aligned with progressive reductions in standardized rating scale scores that were systematically assessed throughout the treatment period. By the completion of the 27‐session protocol on 11 June 2024, the patient showed substantial reductions in symptom severity across multiple domains: the Hamilton Depression Rating Scale (HAM‐D‐17) decreased from a baseline score of 26 to 4, the Hamilton Anxiety Rating Scale (HAM‐A) fell from 34 to 6, and the Pittsburgh Sleep Quality Index (PSQI) improved from 16 to 5. Concurrently, office blood pressure readings stabilized in the normal range (125–135/80–85 mmHg) compared with pre‐treatment values that frequently exceeded 180/110 mmHg. Hemolacria frequency, based on patient self‐report, reduced from 4 to 5 episodes per day to one minor unilateral episode approximately every 43 days (Figure 3).

These observations demonstrate that substantial reductions in psychiatric symptoms, blood pressure, and hemolacria frequency occurred in temporal association with anodal tDCS targeting the left dorsolateral prefrontal cortex, without any change in concomitant medications. No adverse effects were reported at any session.

At the three‐month follow‐up evaluation in September 2024, all observed improvements were sustained without any additional tDCS sessions or changes to the pharmacotherapy regimen. The HAM‐D‐17 score was 3, HAM‐A score was 5, and PSQI score was 4; blood pressure remained stable at 128–132/78–84 mmHg; and no further episodes of hemolacria were reported during the entire post‐treatment interval.

These findings should be interpreted as hypothesis‐generating. Given the single‐case, open‐label, uncontrolled design of this report, causality cannot be established, and alternative explanations including placebo response, non‐specific effects of intensive clinical attention, regression to the mean, or spontaneous remission cannot be excluded.

## 5 Discussion

The present case describes a patient with a complex clinical presentation characterized by treatment‐resistant major depressive disorder, generalized anxiety disorder, insomnia disorder, refractory hypertension, and recurrent bilateral hemolacria of probable functional origin. Following 27 sessions of anodal tDCS targeting the L‐DLPFC, marked reductions in symptom severity across all domains were observed in temporal association with the intervention. To our knowledge, this represents the first published report of tDCS application in a patient presenting with hemolacria.

Several mechanisms may plausibly link improvement in psychiatric symptoms with reduced hemolacria frequency. Chronic severe stress and depression are associated with heightened sympathetic tone and elevated circulating catecholamines, which can increase microvascular permeability, including in the lacrimal gland [3, 6]. Anodal tDCS of the L‐DLPFC has been shown in some studies to reduce sympathetic outflow and improve autonomic regulation. These centrally mediated effects could, in theory, influence lacrimal microvasculature via descending autonomic projections; however, this pathway remains entirely speculative. A more parsimonious explanation is that the observed reduction in psychiatric symptom severity led to decreased sympathetic hyperarousal, with secondary improvement in hemolacria frequency. The concept of a “brain–eye–vascular triad” described in the low vision and blindness rehabilitation literature [15] is not directly applicable to the present case and should be considered only as a loose analogy.

The patient's history of two severe traumatic brain injuries (TBI)—including a six‐month coma and small bilateral frontal contusions—represents a critical and under‐developed aspect of this case. Post‐TBI autonomic dysregulation, paroxysmal sympathetic hyperactivity, and altered prefrontal–autonomic connectivity are well documented and could have contributed significantly to both the refractory hypertension and stress‐related somatic manifestations such as hemolacria. The overall clinical syndrome may therefore be best conceptualized within a framework of post‐TBI affective and autonomic dysregulation, in which prefrontal dysfunction plays a central role. In this context, anodal tDCS targeting the left DLPFC may have exerted its effects partly by modulating residual prefrontal regulatory capacity over autonomic networks. However, the extent to which prior TBI influenced the neurophysiological response to tDCS remains unknown, and the present findings may not generalize to individuals with functional hemolacria without a comparable history of significant brain trauma.

Hemolacria is a rare and highly distressing condition that mandates systematic exclusion of organic causes before psychosomatic or functional mechanisms are invoked [1, 2]. In the present case, exhaustive investigations revealed no local ocular, lacrimal, vascular, or systemic abnormality. This profile aligns with earlier reports in which hemolacria resolved after successful treatment of severe anxiety or depression [8].

Key limitations must be acknowledged. This is an uncontrolled single‐case observation; causality cannot be inferred. Importantly, the primary somatic outcome (hemolacria frequency) relied almost exclusively on patient self‐report via a daily diary, with no episodes directly witnessed by clinicians. The intensive clinical contact inherent to the tDCS protocol may itself have conferred non‐specific therapeutic benefit. Furthermore, even though medications had been stable for at least six months, delayed or cumulative effects cannot be entirely ruled out. The three‐month follow‐up is relatively brief, limiting conclusions about long‐term durability. Finally, subtle variations in adherence or pharmacokinetics cannot be excluded.

These findings are therefore hypothesis‐generating and underscore the need for larger, randomized, sham‐controlled trials to establish whether L‐DLPFC tDCS exerts specific therapeutic effects in functional hemolacria and related stress‐associated somatoform disorders, particularly in patients with a history of TBI.

## Author Contributions

**Roghayeh Mohammadi:** conceptualization, data curation, formal analysis, funding acquisition, investigation, methodology, project administration, writing – original draft, writing – review and editing. **Ahmad Alipour:** resources, software, supervision, validation, visualization.

## Funding

The authors have nothing to report.

## Ethics Statement

The study was approved by the Ethics Committee of Payame Noor University.

## Consent

Written informed consent was obtained from the patient for publication of this case report and accompanying images.

## Conflicts of Interest

The authors declare no conflicts of interest.

## Data Availability

The datasets generated during and/or analyzed during the current study are available from the corresponding author on reasonable request.
